# The Potential Use of the CRISPR-Cas System for HIV-1 Gene Therapy

**DOI:** 10.1155/2019/8458263

**Published:** 2019-08-21

**Authors:** Gabriela De Nardi Sanches-da-Silva, Luiza Fonseca Sales Medeiros, Fabio Mitsuo Lima

**Affiliations:** Centro Universitário São Camilo, Avenida Nazaré, 1501, São Paulo, SP, CEP 04263-200, Brazil

## Abstract

The HIV-1 virus (human immunodeficiency virus) affects 36.9 million people worldwide, with approximately 900000 deaths in 2017. The virus carrier can develop severe immunodeficiency since CD4^+^ T lymphocytes are the main target, leading to acquired immunodeficiency syndrome (AIDS). Despite advances in pharmacological treatment, it is still difficult to eliminate latent reservoirs, becoming one of the main obstacles for viral eradication. The CRISPR- (clustered regularly interspaced short palindromic repeat-) Cas system is a genome-editing method which uses a guide RNA, a complementary sequence to the interested site, recruiting a nuclease that can break the viral or the host cell genetic material. From this double-stranded break, cellular repair mechanisms are activated being able to generate deletions, insertions, or substitutions, in order to inactivate specific gene loci, leading to loss of function. The objective of this minireview is to synthesize the current knowledge on the application of CRISPR-Cas-based gene therapy for HIV-1. The strategies encompass all steps of the viral infection cycle, from inhibition of cell invasion, through viral replication and integration inhibition, to excision of the latent provirus. Off-target effects and ethical implications were also discussed to evaluate the safety of the approach and viability of its application in humans, respectively. Although preclinical and clinical tests are still needed, the recent results establish an exciting possibility of applying this technology for prophylaxis and treatment of HIV-1.

## 1. Introduction

The human immunodeficiency virus (HIV-1), the etiological agent of acquired immunodeficiency syndrome (AIDS), is an enveloped lentivirus formed by two single-strand RNA molecules, wrapped by a capsid [1]. The viral genome is composed of a long terminal repeat (LTR), located at both ends of the molecule and by nine overlapping genes called *gag*, *pol*, *env*, *tat*, *rev*, *nef*, *vif*, *vpr*, and vpu [1]. The main virus transmission sources are the unprotected sexual contact, contaminated blood, sharing of contaminated syringes and needles, and vertical transmission [2, 3]. It is estimated that in 2017, worldwide, 36.9 million people were living with HIV, with approximately 900000 deaths and 1.8 million new infections [4]. The most severe symptoms caused by HIV infection are due to other opportunist infections, most of which are more intense due to acquired immunodeficiency. However, a large number of people carrying HIV-1 may show early symptoms (around 2-6 weeks after the infection) similar to the flu that is called retroviral syndrome that oftentimes goes unnoticed [5].

Despite the high incidence and prevalence of the disease, many efforts have been made over the last decades, with particular attention to antiretroviral therapy (ART) to increase survival and reduce hospital admissions, complications due to opportunistic pathogens, and mortality [3, 6]. However, pharmacological therapy is not completely competent to promote cure owing to the persistence of the virus in latent reservoirs, which includes macrophages, microglia, astrocytes, intestinal lymphoid cells, and, mainly, CD4^+^ memory lymphocytes and failure of patient adhesion to treatment [7–9]. Thus, new therapeutic approaches are necessary. Gene therapy based on the CRISPR-Cas genome editor emerges as a powerful tool to interfere at different stages of the virus infection cycle in the host, from preventing virus entrance into the cell to excising the provirus from those infected [10, 11].

The aim of this review is to summarize information about how CRISPR-Cas-based gene therapy can target all steps of the viral infection cycle and how it can be helpful for the treatment of HIV-1 patients in the near future.

## 2. Summary of the HIV-1 Infection Cycle

**Figure 1:**
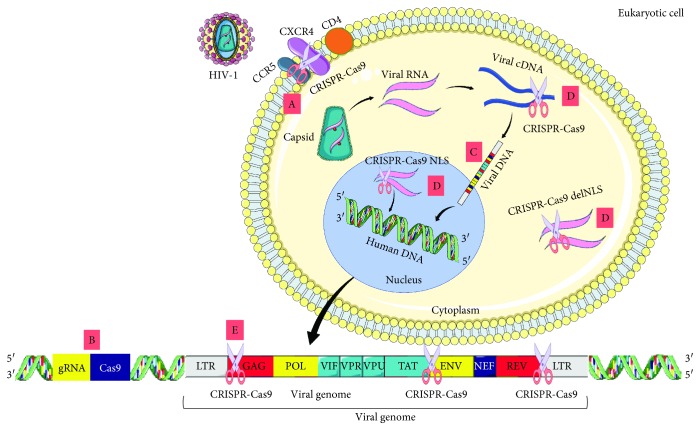
Overview of the CRISPR-Cas strategy to interfere on the HIV-1 infection cycle. A: use of the CRISPR-Cas system to introduce loss-of-function mutations in the CCR5 and/or CXCR4 coreceptors in several cell types; B: inhibition of virus-cell invasion, reverse transcription, and integration by Cas and gRNA stable expression from the host cell genome; C: inhibition of viral replication through targeting gRNAs to different sites in the HIV-1 genome, including LTR, *gag*, *pol*, *tat*, and *rev*; D: inactivation of viral genetic material prior to integration into host DNA by transductions with Cas9-NLS or Cas9-delNLS and gRNA, whose targets are the R and U5 regions of LTR; E: rupture of the proviral genome from latent reservoirs with the LTR region as the main target, or by targeting other viral genes, thus modulating several HIV-1 characteristics and its infectious capacity.

Once inside the host, HIV-1 crosses the mucosal barrier and binds to CD4^+^ receptors present in macrophages, dendritic cells, and CD4^+^ T lymphocytes by using gp120. This event promotes conformational changes in the glycoprotein, facilitating the binding to the chemokine coreceptors CCR5 or CXCR4 (Figure 1), the first one being expressed in the CD4^+^ T cells and the second in the others. After this association, viral gp41 fuses to the target cell membrane and integration of viral and cellular membranes takes place, leading to the release of the capsid into the cytoplasm (Figure 1). Then proteases will act on capsid, releasing viral RNA [12, 13].

In the cytoplasm, reverse transcriptase from HIV-1 uses the viral single-stranded RNA as a template, giving rise to double-stranded viral DNA, which will be inserted into the host chromosome by the virus integrase (Figure 1). Later, the integrated HIV-1 genome will be transcribed and translated. After the production of viral proteins and replication of its genetic material, the assembly of new viruses begins, which will include part of the host cell membrane to form the envelope in a process known as “budding off,” releasing mature and infectious viral particles [12]. For an in-depth review of the HIV-1 infection cycle, refer to other articles [14–16].

## 3. HIV *env* Gene and Coreceptors

The *env* gene encodes viral envelope glycoprotein gp160, precursor of gp41 and gp120 glycoproteins. The latter extends outside the viral lipid membrane, and its main function is to bind to the host cell receptor, determining its tropism. In addition, gp120 presents multiple recognition sites for several adaptive immune responses. It was broadly categorized into five hypervariable regions (V1 to V5) with conserved interspersed regions. Thus, two positive selective forces act on the gene *env*: (1) to alter the optimal affinity to the host cell receptor and (2) to evade of host immune responses [17, 18].

As mentioned above, it is known that for HIV-1 host cell invasion, viral binding to the CD4 molecule is required together with coreceptors CCR5 and CXCR4. The tropism of HIV, therefore, is largely due to the expression patterns of these two coreceptors [19].

There are two types of HIV strains, the T cell tropics and the macrophagic (tropism M). The latter uses CCR5 as the coreceptor in the infection of macrophages and primary T cells and involves 90% of the primary infections. They are the most common viruses isolated from asymptomatic individuals, typically being transmitted between humans. On the other hand, T-tropic viruses can evolve throughout the disease due to mutations in the envelope protein. This strain uses CXCR4 as a coreceptor [18–20]. However, it is important to note that there are evolutionary dual-tropic viruses that can infect cells expressing CXCR4 or CCR5 [19].

Some individuals are highly resistant to HIV infection, but not completely immune to it. They have a 32 base pair deletion in the CCR5 gene, causing a frameshift and creating a protein that does not reach the cell surface. This contributed to prove the importance of this coreceptor, since it has been observed that the levels of the coreceptor are correlated with degree of infection. Therefore, even if virus-infected individuals are heterozygous for this mutation, there will be a survival advantage compared to nonmutated individuals as they express less CCR5 in their cells, which delays HIV replication and, consequently, the death of the T CD4^+^ CCR5^+^ lymphocyte. Virus entry into the CCR5-mediated host cell may limit the infection, even in patients with a single copy of the gene [18, 19].

## 4. Principle of the CRIPSR-Cas Technology

In 1995, Mojica et al. reported the identification in archaea chromosome of long stretches of 30 bp tandem repeats (TREPs) interspersed with up to a 39 bp unique sequence [21]. It was followed by the characterization of these chromosomal regions and the first studies to understand their biological function [22, 23]. Subsequently, these repeats were renamed to CRISPR (clustered regularly interspaced short palindromic repeat) and Cas genes (CRISPR associated) were identified as adjacent to it [23]. In addition, the CRISPR locus was found to be transcribed and processed into small RNA fragments, which could present several functions such as resistance to bacteriophage [24].

**Figure 2:**
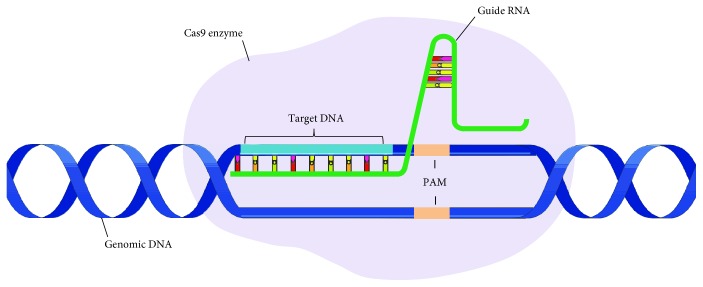
Principle of the CRISPR-Cas technology. A guide RNA (green) is composed of a crRNA sequence that is specific to a target DNA (light-blue), linked to a tracrRNA sequence that interacts with Cas endonuclease. The complex recognizes the PAM sequence (protospacer adjacent motif), a trinucleotide immediately following the targeted DNA to orientate its cleavage. Genomic DNA is showed in dark blue.

Ten years after the first findings, the relationship between CRISPR and bacterial immunity was established, from the hypothesis that the unique sequence cited above was from extrachromosomal origin as plasmids or bacteriophage. Thus, an invader would not be able to infect bacteria with specific spacers against it [25, 26]. It was observed that transcription of CRISPR locus gives rise to CRISPR-derived RNAs (crRNAs) that were thought to target foreign DNA by complementarity [27, 28]. Another transcribed RNA is called trans-activating crRNA (tracrRNA) which is a small RNA with 24-nucleotide complementarity to crRNA precursor transcripts [29] that direct crRNA maturation to protect the host from exogenous DNA (Figure 2).

In 2012, Jinek et al. revolutionized gene therapy by presenting the junction of crRNA to tracrRNA forming a single RNA strand, known as single-guide RNA (sgRNA) [30], capable of guiding and activating Cas9 to break targeted DNA (Figure 2) [30, 31]. This break is dependent on the presence of a three-nucleotide sequence called protospacer adjacent motif (PAM) [32]. In this way, it was verified that the CRISPR-Cas system can be used to activate or inhibit genes [33]. From this, numerous studies were performed using this genomic editor for different applications. One of the more prominent is its use in gene therapy for HIV-1 as we discuss below.

## 5. CRISPR-Cas for CCR5 Interruption

An important intervention involves CCR5 coreceptor interruption, which has been shown to be one of the main targets for drug and gene therapy against virus infection [34, 35] (Figure 1, A). This chemokine receptor is associated with G protein whose ligands are proinflammatory cytokines (CCL3, CCL4, and CCL5) and play an important role as a costimulatory molecule in immunological synapses [34, 36].

This is considered the main coreceptor for R5 tropic HIV entrance into cells, especially those transmitted by sexual contact, maternal-infant exposure, and percutaneous inoculation. Therefore, it plays a crucial role in the onset of viral infection, which has led some authors to test alternatives to induce changes in the gene encoding this cell surface protein. Similar proposals were given by Wang et al. [35] and Li et al. [37]. Transduction of the CRISPR-Cas9 system occurred in TZM.bl cells susceptible to HIV-1, which express the CCR5 and CXCR4 coreceptors [35, 37]. In the first work, three gRNAs were used to target the CCR5 gene, namely, CR1, CR2, and CR3 [35]. On the other hand, Li et al. [37] used eight gRNAs, known as sgR5-3 to sgR5-10. Wang et al. [35] observed that, after seven days, the percentage of negative cells for CCR5 expression on the cell surface was 10.8%, 67.7%, and 36.7% for CR1, CR2, and CR3, respectively (Table 1). The results obtained by Li et al. [37] indicated that sgRNA-5 and sgRNA-8 induced the most significant effects, resulting in 74.1% and 63.8%, respectively, of the mutations in the allele of the CCR5 gene, leading to decreasing in protein expression [35, 37] (Table 1).

To confirm the intervention success, both authors infected their cell groups with HIV-1 pseudotypes and found resistance to postedition HIV-1 infection for pseudotypes with R5 tropism. However, it does not indicate protection to the virus with R4 tropism [35, 37]. In addition, the effect of editing was assessed on primary CD4^+^ T cells and susceptibility to HIV-1 infection markedly declined [37].

This may indicate that because the CCR5 coreceptor is essential for R5 tropic virus entry, its partial ablation may provide clinical benefits for HIV-1 patients. In fact, changes in its expression are becoming effective *in vivo* when modified cells are transplanted into an animal model. Xu et al. [38] used NPG rats, nonobese animals, and immunodeficient diabetics, who were previously transplanted with hematopoietic stem and progenitor cells (HSPCs) containing CCR5 or nonedited ablation. It was observed that, after being challenged with an R5 tropic strain, animals that received edited cells showed a reduction of viral RNA levels in the peripheral blood, after fifteen days of infection [38] (Table 1).

Due to the mentioned events, after transplantation, rapid and efficient hematopoietic reconstitution was observed and edited cells were detected twelve weeks after the procedure. Therefore, experimental evidences confirm the promising *in vivo* approach that transplantation of cells edited for CCR5 would aid in HIV-1 therapy [37, 38].

The natural-occurring 32-base pair deletion in the CCR5 gene (CCR5*Δ*32) generates a stop codon, and consequently, the absence of expression on the cell surface leads to slower progression or resistance to HIV-1 infection with R5 tropism [39–41]. This polymorphism apparently does not influence the susceptibility to other virus infections as observed for HCV [42] and influenza A (H1N1) [43].

Inducing this deletion using CRISPR-Cas9 is another approach, foremost reported by Ye et al. [44], which used human-induced hematopoietic stem cells cotransfected with Cas9 and gRNA to target CCR5, leading to the generation of biallelic or monoallelic CCR5*Δ*32 mutation [44]. Confirmation of resistance was observed in cells that differentiate into monocytes and macrophages, and after inoculation with R5 tropic HIV-1 virus, beneficial results were obtained by the reduction of viral replication compared to nonaltered cells [44]. Some years later, this alteration was tested using primary Jurkat and CD4^+^ T cells from peripheral blood mononuclear cells. Qi et al. [41] used a lentiviral vector with Cas9 and two gRNAs targeting the CCR5*Δ*32 locus. The analysis indicated the efficiency of the genomic edition for all possibilities tested in Jurkat cells, in which about 60% of the CCR5 mutations were given by the *Δ*32 deletion. In CD4^+^ T cells, this mutation in the coreceptor occurred in 20% of the cells [41] (Table 1).

The knowledge about this natural mutation and its relevance to viral entry made the transplant and this therapy promising for HIV patients [44, 45]. A recent publication by Gupta et al. [45] indicated the reduction of viral RNA to undetectable levels in HIV-positive patient with Hodgkin's lymphoma, subjected to an allogeneic transplantation of hematopoietic stem cells from a donor carrying biallelic CCR5*Δ*32 mutation [45].

This patient was regularly monitored, and CD4^+^ and CD8^+^ T lymphocytes without CCR5 expression were collected, suspending antiretroviral use 510 days after transplant. Over again, confirmation was performed after infection with CCR5 or CXCR4 tropic HIV, and as expected, cells from the donor were not infected with R5 strains but with X4 trophic [45]. Previously, a similar outcome was presented by Hütter et al. [46] in a HIV-1 positive patient with acute myeloid leukemia, who also retained undetectable viral load during the posttransplant analysis of cells with homozygous mutation for the CCR5 allele, indicating the central role of this coreceptor [46] (Table 1).

Therefore, data confirm the important and promising approach that *in vivo* transplantation of cells edited for CCR5 would aid in HIV-1 therapy. Although the ART treatment can select the R5X4 or X4 resistant, the CRISPR-Cas9 system may indicate an efficient molecular tool for the near future [37, 38].

## 6. CRISPR-Cas for CXCR4 Interruption

The CXCR4 coreceptor is a G protein-coupled chemokine receptor, important for controlling migration to the chemokine CXCL12 gradient, which is important for the retention of hematopoietic stem cells in bone marrow [13, 47, 48]. At the HIV-1 context, as described earlier, CXCR4 is used by the virus to enter the cell with X4 tropism, and in the late infection process, R5 tropism strains can transform into double-tropism strains, using both coreceptors to enter the cell [13, 49]. In this way, its interruption is an interesting strategy to avoid infection (Figure 1, A). Hou et al. [48] have recently used a lentivirus expressing Cas9 and 10 different gRNAs orientated to conserved sites of the CXCR4 gene to introduce functional loss mutations. The authors used osteosarcoma-derived cells, Jurkat T cells, and infected primary human CD4^+^ T cells. The results showed an efficient edition in all tested cell lines, mainly for two gRNA targets, leading to alterations in the CXCR4 expression level (Table 1). It was observed that the number of cells expressing the protein reduced to 23.5% and 29.9% in osteosarcoma-derived cells for these gRNAs. However, this same technique suggested less cleavage efficiency in human CD4^+^ T cells. This strategy induced HIV-1 resistance for edited cells [48].

A similar evaluation was performed by Schumann et al. [50], who used Cas9 ribonucleoproteins to edit primary human CD4^+^ T cells isolated from healthy donors. The intention was to induce indels in the CXCR4 gene. An exogenous template for homology-directed repair (HDR) was introduced to replace 12 nucleotides from the original sequence in four different concentrations. The results indicated that 60% of cells reduced the CXCR4 expression on the cell surface [50] (Table 1). Together, these findings indicate that this approach could be useful for the generation of experimental and therapeutic primary human CD4^+^ T cells, providing an alternative way to treat HIV-1 X4 infection [50].

On the other hand, it is important to consider the coreceptor relevance in hematopoietic cells, where changes in CXCR4 expression could prejudice its physiology. For this reason, the authors certified that the Cas9-mediated CXCR4 ablation was highly specific, with an insignificant effect on cell division and propagation. In addition, treated human CD4^+^ T cells have apparently immune functions preserved [48]. In line with this, Liu et al. [49] induced CXCR4 P191A mutation in TZM.bl cells combining CRISPR-Cas9 and the *piggyBac* transposon system [49]. It was observed that natural CXCR4 mutant P191A precludes HIV-1 attachment but retains its physiological function [51]. The authors demonstrated a reduction in HIV-1 infection by decreasing the CXCR4-positive cell population from 99.8% to 18.4% and 12.0% after treatment with two different sgRNAs [49]. Therefore, this is a complementary strategy to inhibit HIV-1 infection, especially in patients with progression to chronic disease [48].

## 7. CRISPR-Cas and Simultaneous Interruption of CCR5 and CXCR4

A more complex strategy is to simultaneously disrupt both coreceptors CCR5 and CXCR4 (Figure 1, A). Liu et al. [52] demonstrated this approach using TZM.bl cells, Jurkat T cells, and primary CD4^+^ T cells. The results indicated that in TZM.bl cells, the indel mutation rate was up to 40.5% for CXCR4 and up to 32.9% for CCR5 [52]. In Jurkat T cells, they also showed the coreceptor disruption and the presence of indels, whereas in the CD4^+^ T lymphocytes, despite the low efficiency as indicated by previous data, there was a significant alteration in the gene structure of the coreceptors, not influencing apoptosis or causing cellular toxicity. In addition, it was indicated that all cells reduced CCR5 and CXCR4 expression, besides becoming resistant to the infection by tropic viruses R5 and X4, even when using double-tropism virus [52] (Table 1).

## 8. CRISPR-Cas to Inhibit Viral Infection

As mentioned above, HIV-1 infection is a multistep process, comprising the virus-cell invasion, reverse transcription of RNA molecules, and its integration into the genetic material of the host cell [53] (Figure 1, B). One possible alternative to inhibiting this cycle is to generate a prophylactic stable immunity against lentivirus infection by expressing Cas9 and gRNA constitutively. Liao et al. [54] analyzed the viral infection in a human T cell line derived from lymphoblasts for a period of fourteen days. Guide RNA was targeted to different positions of the HIV-1 genome. The results indicated a persistent reduction of viral expression in cells harboring HIV-1-targeted gRNA, especially for the R and U3 regions present in LTR [54]. Similar results were observed using CD4^+^ T cells acquired from five different donors. The results confirmed the reduction of HIV-1 production by more than three times in relation to the control groups (Table 1). These findings suggested that these lines developed protection against viral infections similar to those obtained by means of transient transduction and could remain for a long period of time [54].

The same application of the CRISPR-Cas9 system occurred in other hematopoietic lines that serve as reservoirs of HIV-1, such as monocyte and macrophage. Several anti-HIV human pluripotent stem cell (hPSC) lines were generated containing a stable expression of Cas9. Cells that would be differentiated into monocyte-macrophage were separated and infected with HIV-1 M-trophic virus. After three days, it was observed that these cells acquired resistance to HIV-1. An important issue was the confirmation that this system does not cause genotoxicity since hPSC undergoes several multiplications and differentiation processes before maturation. Furthermore, the most efficient target for the LTR region, known as LTR-T2, did not induce off-target effects since this region presents similarity to human genome sequences [54].

The following year, Kaminski et al. [55] adopted an elegant strategy by placing the gene encoding Cas9 under the control of a promoter activated by viral Tat. They evaluated whether viral Tat released in the infection process could stimulate the constitutive nuclease production in the cell, since this is important for HIV-1 transcription [55]. TZM.bI cells were treated with an expression vector containing specific regions of the HIV-1 promoter and sequence for Cas9, then receiving multiplexed gRNAs A and B targeted to the LTR region [55]. After being infected with different amounts of HIV-1, the results indicated cleavage of the viral genetic material, suggesting that the production of Tat during the infectious process stimulated the promoter for the Cas9 expression, promoting HIV-1 ablation in the initial stage or reactivation of latent virus [55] (Table 1).

Previously, Hu and colleagues [56] used the CRISPR-Cas system as prophylaxis against virus infection based on the use of TZM.bI cells containing Cas9 and gRNAs A and B, directed to the LTR region in a constitutive manner [56]. Cells were infected with different HIV-1 strains expressing GFP, indicating competent replication. The authors observed that stable expression of Cas9 and gRNAs prevented a recent viral infection and consequently immunized the cells. In addition, cell growth and viability remained equivalent to controls, without toxicity or off-target effects, assessed by gRNA specificity to its target and by sequencing the complete genome of the TZM.bI cells [56]. Taken together, these studies demonstrate that the use of the CRISPR-Cas system as a vaccine would be an important strategy, acting independently of the HIV-1 strains present in the infection, since the targets are viral genomic sequences and may act prior to integration into the genetic material of the host cell (Figure 1, B) [56] (Table 1).

## 9. CRISPR-Cas to Inhibit Viral Replication

Another strategy that has been studied is the inhibition of viral replication (Figure 1, C). Thus, gRNAs were targeted for different sites present in the HIV-1 genome, including LTR, *gag*, *pol*, *tat*, and *rev*. This event could occur in the cytoplasm or inside the nucleus. The first strategy to prevent HIV-1 replication was established by Yin et al. [57]. The authors used HEK293T cells transfected with a plasmid carrying Cas9 with a nuclear location signal. In addition, these cells received multiplexed gRNAs, one directed to the LTR region and others to the *gag* or *pol* genes, in order to obtain the best combination [57]. The results indicated high efficiency in all combinations involving gRNA for the *gag* and LTR genes, reducing luciferase expression by up to 96%. The use of gRNA against LTR with any other targets reduced the protein by up to 23%. Thus, the combination of gRNAs targeting the LTR region and structural genes is an important strategy for accurately targeting the virus [57] (Table 1).

Using a similar approach, Yin et al. [53] observed a reduction of 57-89% in the expression of the gene reporter as well as Gag expression. Guide RNAs targeted to LTR and *tat* induced a greater reduction in expression in relation to the other genes [53] (Table 1). Noteworthy is that Cas9 endonuclease can be targeted to several sites where the HIV-1 genetic material is present. Therefore, the use of a modified Cas9 enzyme, which lacks the nuclear location signal, is important to keep the enzyme in the cytoplasm. This could be a solution to reach viral DNA earlier (Figure 1, D) while using the Cas9-NLS enzyme would be the possible target HIV-1 genome in both cell compartments [53] (Figure 1, D).

The viral replication suppression, mediated by CRISPR-Cas, was also observed in CD4^+^ T cells extracted from healthy patients. Primary cultures were infected with HIV-1 and subsequently with the lentivirus vector delivering Cas9 and gRNAs A and B targeted to the LTR region. There was a reduction in the number of copies of HIV-1 present in the treated cells [55] (Table 1).

Similar analysis using peripheral blood mononuclear cells (PBMC) and CD4^+^ T cells from four seropositive patients submitted to ART treatment showed a decrease in viral cDNA number in the order of 81% and 91% for PBMC in two patients [55]. As for CD4^+^ T cells, the reduction was of 92% and 56% in two individuals [55]. As a result, there was a reduction in the number of viral particles and expression of Gag proteins and p24 in all cases [55]. As expected, the number of copies was reduced by the advent of indels and single-nucleotide variations (SNVs) at or near the protospacer adjacent motif (PAM) present in the target regions [55] (Table 1).

The inhibition strategy of HIV-1 viral replication was also adopted by Wang et al. [58] and Lebbink et al. [59] who used Sup-T1 cells treated with two lentiviral vectors with Cas9 and gRNAs against different viral genome targets, including the LTR region and several genes [58, 59].

The first study monitored the HIV-1 replication by the presence of p24 in culture. Cells treated with Cas9 and gRNAs showed reduced expression of this protein, especially those that received gRNA targeted to conserved regions of the virus genome, obtaining a vigorous decline relative to those directed to less conserved targets. This fact could contribute to viral escape [58]. The second article indicated that the set of two gRNAs being considered strong could completely abrogate viral replication, different from those in which the gRNA pair was less effective, which generated a partial control of the infection process and viral progression [59] (Table 1).

In addition to previous findings using Sup-T1 cells, Wang et al. [60] analyzed whether the constitutive use in the Cas9 and T4 or T10 gRNAs, targeting to read frames between *gag*-*pol* and e*nv-rev* genes, respectively, could inhibit viral replication and prevent HIV-1 escape [60].

Moreover, the results demonstrated that even in a short period of infection, there was a reduction in the number of HIV-1-infected cells and in the genesis of infectious particles, caused by indels in the target regions. As a consequence, there was a decline in reverse transcriptase activity due to reduced viral replication [60]. However, the authors emphasize the importance of generating some indels, since these can be lethal to the virus, while others can generate beneficial recombination for their infectivity and resistance. This indicates that if the target is not effective to inhibit, for example, viral replication, the changes caused in its genome may lead to adaptive improvement. Therefore, the use of multiple gRNAs for different HIV-1 targets would be an option to avoid these events [60] (Table 1).

## 10. CRISPR-Cas to Prevent Viral Integration

In addition to the strategies described until now in this review, some authors have attempted to break the HIV-1 genome prior to its integration into the host DNA. Liao et al. [54] and Yin et al. [53] tested, *in vitro*, whether the HIV-1 genetic material could be cleaved and degraded in the cytoplasm (Figure 1, D). The first authors evaluated whether the synthesized HIV-1 complementary DNA (cDNA), when released inside the host cell, would be cleaved by Cas9, preventing its infection and integration. Using a GFP reporter gene, it was possible to observe a significant reduction of positive cells [54]. The second authors, for a similar purpose, used HIV-1-infected 293T cells. These were transduced with Cas9-NLS and gRNA, whose targets were the R and U5 regions of LTR. The amount of viral synthesized DNA including early, late, and integrated was analyzed. Three- to fivefold reduction of integrated viral DNA was observed: twofold reduction for late DNA (preintegrated) and no significant change for early (cytoplasmic) DNA products. One reason for early DNA to remain unchanged is that Cas9 had a nuclear location signal, and these early products of reverse transcription are mainly present in the cytoplasm. As a result, it was possible to analyze that the CRISPR-Cas9 system can not only inactivate integrated HIV-1 because of the indels but can also reduce the number of proviruses due to the degradation of its genetic material prior to integration [53].

## 11. CRISPR-Cas and Latently Infected Cells

Although very promising, the strategies described above use CRISPR-Cas9-based gene therapy for prophylaxis against viral infection. However, to cure patients who carry the provirus in the latent form is the ultimate goal of most researchers in the area (Figure 1, E). Some alternatives have already been proposed to promote the removal of latent provirus; however, this remains a challenge. Two strategies stand out: the first, with LTR as a target, promotes the simultaneous cleavage of the two regions, removing an internal portion of the proviral DNA present in the genome host. The second alternative is to act on viral genes, allowing to modify several characteristics of HIV-1 and its infectious power [54, 61].

The first demonstrations of this potential were given by Ebina et al. [61]. For this, 293T and HeLa cells were infected with pseudotyped HIV-1. After infection, some cells received plasmid containing gRNA targeting for T5, which is present in the TAR sequence of the R region, while other cells received gRNA targeting for T6 present in the NF-*κ*B site sequence of the U3 region. Then all cells received a second plasmid expressing Cas9. Analysis of the results was given by the expression of the GFP reporter gene. The results showed that in 293T cells, there was a greater reduction of the expression especially in those that received the T5 gRNA, reducing expression from 45.6% to 20%. In HeLa cells, a small reduction in GFP expression was observed due to lower transduction efficiency [62] (Table 1). It is important to emphasize that the success is due to the TAR region being relatively conserved and when cleaved, complex formation to stimulate viral transcription becomes critical. The altered fragments of the LTR were isolated, and the results confirmed the presence of several mutations at the T5 cleavage site, including deletions, insertions, or combinations between them, as a consequence of the nonhomologous end joining mechanism (NHEJ). These were responsible for the alteration in viral transcription [61].

Two years later, Liao et al. [54] performed similar experiments, but with HEK293T cells containing different amounts of integrated viral DNA. For this purpose, gRNA was constructed for both the LTR region and different sites from the GFP coding region. The results, after fourteen days of infection, confirmed the potential of this editor, in which regardless of the amount of integrated material, there was a reduction in the expression of this protein [54] (Table 1). The efficiency increased according to the number of infections, and again, this change occurred by means of indels established at the target sites, as observed earlier [54, 61]. This suggests that a possible chronic low-dose treatment could be effective in eradicating the provirus over time and would cause low cytotoxicity [54].

The attempt of many authors to generate the rupture of the provirus genome has the objective to compromise a possible reactivation of it. This is particularly problematic in HIV-1 positive patients who discontinue ART. In order to evaluate whether, after treatment with CRISPR-Cas9, reactivation of the provirus was suppressed, Zhu et al. [63] used Jurkat cells containing integrated HIV-1 DNA in its genome. These were initially treated with Cas9 and gRNAs targeted to different viral region, including LTR and *pol, tat* and *rev* genes. TNF-*α* was provided for viral gene expression activation, producing GFP and p24. The authors observed up to 10-fold GFP reduction as well as up to 20-fold p24 reduction, according to the gRNA used. In addition, the possibility of using multiplex gRNA, in different combinations, potentiated the reduction in HIV-1 expression by up to 24-fold, especially for *tat* and r*ev* [63] (Table 1).

Complementarily, Wang et al. [62] confirmed the findings in the c11 lineage from Jurkat cells transduced with lentivirus containing Cas9 and gRNA for the LTR region. These were submitted to suberoylanilide hydroxamic acid (SAHA), a histone deacetylase inhibitor, to reactivate the provirus. It was observed that the viral reactivation was low, being of 4.5% of cells positive for expression of the reporter gene in relation to 25.9% expression in control cells after SAHA treatment [62] (Table 1). These results demonstrate the great potential of this strategy in the treatment of chronic patients (Table 1).

In addition, other cell types that are important reservoirs, such as myeloid cells and astrocytes, can also be effectively altered by CRISPR-Cas9. Studies based on these cells are important since HIV-1 can persist in the central nervous system (CNS), being a great challenge for ART [64].

The seminal paper from Hu et al. [56] demonstrated for the first time that the CRISPR-Cas system could eradicate HIV-1 proviruses. The authors used a latent HIV-1 myeloid cell model treated with Cas9 and four possible gRNAs targeted to the U3 region of LTR, designated as A to D. These cells then received the histone deacetylase inhibitor TSA to activate the transcription of the integrated proviruses. The results indicated a significant reduction in GFP expression in treated cells [56]. Afterwards, multiplexed gRNAs were used, resulting in complete deletion of the proviral fragment, between 5′LTR and 3′LTR. In addition, this strategy generated several indels which led to complete inhibition of viral reactivation and replication [56] (Table 1).

Meanwhile, Kunze et al. [64] verified whether CRISPR-Cas9 would affect viral expression in HNSC.100 cells, an astrocyte cell model containing latent provirus [64]. For this, the cells were infected with an AAv9P1 vector carrying sequences for Cas9 and gRNAs. Latent HIV-1 virus was then activated with TNF-*α* and its expression was evaluated. Quantitative analysis showed a reduction in those cells that received Cas9 and gRNA, indicating success in the edition (Table 1). The authors also observed the presence of indels in the LTR region, which led to the eradication of the provirus, without effects under the cellular genome or cytotoxicity [64].

The second strategy to promote the removal of latent provirus was developed by Liao et al. [54] and Wang et al. [62] who evaluated the efficiency of using more than one gRNA for different targets. Both made a comparison between gRNA targeting structural genes (*gag* and *env*), genes encoding enzymes (*pol*), accessory genes (*vif* and *rev*), and the LTR region. In the first work, it was observed that in HEK293T cells infected with HIV-1 and treated with gRNA for different targets, the authors obtained a 48-92% reduction in GFP expression [54] (Table 1). In the second study, gRNAs were selected against forty-three distinct targets in the HIV-1 genome with low off-target effects on the host. HEK293T cells were transfected with lentivirus containing Cas9 and gRNA. The results indicated that 11 gRNAs for the LTR region and 12 gRNAs for other genes significantly reduced the expression of p24, while others showed little inhibition effect. The eight gRNAs that had the highest inhibitory effects on conserved HIV-1 sequences were selected to reach different sites present in the viral genome and could be evaluated by double transductions [62] (Table 1). The experiments demonstrated that there are different targets in the viral genome that allow its eradication but gRNAs directed to the LTR region were more effective than others [54, 62].

## 12. CRISPR-Cas to Disrupt Integrated Virus from Animal Models

Despite the abundance of results already described *in vitro* on the efficiency of the CRISPR-Cas9 editing system, the introduction of therapeutic genes into living models is still limited. Based on this, some authors have demonstrated the feasibility and efficacy of HIV-1 eradication *in vivo*. Kaminski et al. [65] performed experiments on Tg26 transgenic mice, which contain samples corresponding to the HIV-1 virus integrated into their genome, mimicking viral infection [65–67].

The AAV9 vector was used to deliver the sequences encoding for Cas9 and gRNA targeting to the LTR and *gag* regions. Injections by the tail vein occurred twice, with an interval of five days, and on the fifteenth day, the animals were sacrificed. The analysis was done using DNA extracted from the liver, heart, spleen, lung, kidney, brain, and blood lymphocytes [65].

The results demonstrate, for the first time, the *in vivo* eradication of HIV-1 from various tissues, confirmed by the presence of the same fragments previously found in the *in vitro* test [65]. In addition, the authors analyzed the effect of excision in 32-day rats and observed, in circulating lymphocytes, the removal of HIV-1 genetic material between target regions, indicating proviral eradication [65] (Table 1).

Posteriorly, Yin et al. [68] demonstrated that eradication of HIV-1 provirus was possible in other animal models. The authors used the adenovirus AAV-DJ/8 and gRNA targeted to the same regions used by Kaminski et al. [65], but in a multiplexed form [68]. Initially, conventional NCr animals with no thymus and presenting lymphocytopenia were used. They were infected via retroorbital injection with EcoHIV-eLuc virus and then with AAV-DJ/8, which contained genes for the expression of Cas9 and gRNA [68].

Interestingly, data obtained from longitudinal bioluminescence images, for 19 days, in live mice indicated that viral expression was significantly reduced. The authors demonstrated that the delivery of Cas9 and gRNAs to various organs and tissues was effective and excision of viral genome occurred [68] (Table 1).

Moreover, this efficiency has also been demonstrated in a clinically more relevant living model, humanized bone marrow/liver/thymus (BLT) mice, which are immunodeficient animals formed with fragments of the human liver, thymus, and bone marrow [68, 69]. The results indicated the presence of fragmentary deletion in several organs and tissues, which resulted in the reduction of viral genetic material and, consequently, in the number of proviruses [68] (Table 1).

Recently, Bella et al. [70] used PBMC cells obtained from three HIV-1-positive patients, under ART, and injected the cells into NRG rats. After 1 week, animals were treated with lentivirus containing Cas9 and multiplex gRNA, known as LTR A and LTR B, directed to the LTR region. After two weeks, a reduction above 90% of viral DNA was observed as well as the deletion of a fragment present between the target [70] (Table 1).

Considering the data reported, it is observed that the use of CRISPR-Cas led to excision of the viral genetic material in PBMC cells from human patients under long-term antiretroviral therapy. These aspects also emphasize that the use of multiplex gRNAs attenuate the chances of generating recombinant viruses with beneficial characteristics [70]. The recent results of provirus excision from live animals open the perspective of conducting human clinical trials in the near future [67, 68].

## 13. Off-Target Effect

Specificity remains a major concern for the safety use of gene therapy based on the CRISPR-Cas system. Off-target effect, the DNA breakage at different sites from that previously intended, can cause severe damage to cell physiology and viability.

Several strategies to guarantee Cas specificity have been reported. One possibility remains in the use of high-fidelity endonuclease, which can present mutations in nonspecific DNA contacts [71], which proved to be efficient in reducing the off-target effect. In some strategies, the decrease in the enzymatic activity to reduce off-target effects can lead to loss of on-target activity. Kulcsár et al. [72] produced a highly enhanced fidelity endonuclease which cleaves target DNA only when perfectly matching 20-nucleotide-long spacers are present. Another approach relies on the introduction of a point mutation, namely, p.R691A, which reduced off-target effect but maintained on-target activity [73]. These studies reinforce the importance of using modified nucleases for clinical use of the CRISPR-Cas system.

Furthermore, the development of software tools to aid the selection of target sequences and optimization of the gRNA design is essential for the experimental plan to minimize off-target effects. One of the first initiatives was based on the score of potential off-target genomic locations by bioinformatic screening [74]. Recently, an approach using a machine learning model was developed, where it is possible to predict the potentially best gRNA sequence [75]. In addition, other authors developed, in 2016, the CRISPOR, an algorithm that identify off-target sites caused by a gRNA sequence, comparing with a databank [76]. Together, these studies demonstrate that the gRNA design is currently an optimized stage of the gene therapy.

To further potentiate the targeting of the Cas nuclease to the cleavage site, the approach of chemically modifying guide RNA has been used. An interesting result was obtained by Hendel et al. [77], who modified 5′ and 3′ gRNA termini with 2′-O-methyl, 2′-O-methyl 3′phosphorothioate or 2′-O-methyl 3′thioPACE and observed enhanced efficiency in editing human primary T cells. Another study showed a moderate improvement when 2′-O-methyl 3′phosphorothioate modification is used [78].

One way to decrease off-target effects is to expose the cell to CRISPR-Cas components for the shortest possible time [79]. For this reason, the use of a plasmid coexpressing Cas9 and gRNA, in an unregulated manner, may not be the safest method [80]. The use of ribonucleoproteins can be an alternative to control the edition since patient cell would degrade its components soon after the precise on-target breakage occurs. The efficiency of this approach was observed *in vitro*, since only in the presence of gRNA that RNP could cleave CCR5 gene [81]. Cho et al. also demonstrated that RNP was effective 48 hours after the gRNA transfection and reduced off-target effect was found [82]. As mentioned above, this strategy was adopted by Schumann et al. to generate indels at coreceptor CXCR4 in CD4^+^ T cells from healthy donors to prevent viral invasion [50].

An additional strategy relies on the use of Cas9 nickases. They are modified endonucleases at NHN or RuvC domains that are responsible for DNA break. This modification allows that only one target DNA strand is cleaved, generating single-strand cut. Pioneering studies using paired Cas9 nickases found extensive reduction of off-target events [83, 84] and did not find chromosomal translocations [85]. All these possible plans to avoid off-target effect need to be accompanied by the analysis of the editing site, but preferably the whole genome, to ensure that no unwanted mutation has been generated. For HIV-1, as mentioned in the above sections, the authors demonstrate their concern about this issue. Based on data published so far, it is possible to conclude that no important off-target effect was found after CRISPR-Cas use in provirus genome excision, reiterating the possible applicability of the system in HIV-1 positive patients [56, 83, 86].

For any method validation, especially those based on CRISPR-Cas gene therapy, parameters as precision, specificity, and reproducibility must be extensively proven before clinical use. For HIV patients, including the analysis of their genomic variation and virus sequence inserted therein would be the best way to design gRNA and minimize possible off-target effects.

## 14. Ethical Aspects Involved in Using CRISPR-Cas in Humans

In November 2018, the scientific community was astonished by an announcement of the birth of twins whose embryos were edited using CRISPR-Cas technology to generate immune individuals to HIV-1 infection. He Jiankui, an associate professor at the Southern University of Science and Technology in Shenzhen, China, rekindled, in a way never seen, the discussions and concerns about the use of this gene editor in humans. Despite its great potential and the studies already carried out, including those involving CRISPR-Cas-based gene therapy for HIV-1 reported above, the genomic edition of embryos violates bioethics principles, international consensus guidelines, and national regulations, including Chinese ones [87–89]. The Committee for the International Summit on Human Gene Editing established, among other issues, the need of intensive basic and preclinical studies in accordance with ethical principles and that “modified cells should not be used to establish a pregnancy” [90], both neglected in this episode.

As described in the previous section, basic experiments showed no off-target effects on treated cells or animal models. However, the genomic edition cannot be proven as completely safe for use in humans so far and the harmful effects concerning its use cannot be estimated. Additionally, mosaicism as a result of incomplete edition constitutes a major challenge for researchers.

Even from an ethical point of view, one must consider how individuals generated by genetic edition will be seen and accepted in the society that will be inserted. The consequences of gene editing go beyond the cure of a disease, and this fact cannot be overlooked. The violation committed by He Jiankui raises serious concerns about possible nontherapeutic uses of the CRISPR-Cas system such as eugenics [91], which would be an even more serious consequence of his research.

In this way, we believe that for the appropriate use of this technology in the future, researchers should consider the risks and benefits involved in gene editing in a holistic manner and that regulation and oversight of clinical trials must be strict to combat ideas that violate the bioethical principles.

## 15. Concluding Remarks

Pharmacological therapy for HIV-1 has shown relevant results in recent years, although it is not able to eliminate the latent virus. Thus, new therapeutic alternatives are being developed, and among the most promising ones is the CRISPR-Cas system. Many strategies *in vivo* and *in vitro* have been established to prevent infection and to compromise latent reservoirs. There is also a challenge to combat a highly mutable virus such as HIV-1 using the CRISPR-Cas system, whose effectiveness is largely dependent on how well the gRNA corresponds to the target viral DNA sequence. To address this issue would be the use of a personalized approach, in which the gRNA is designed to match the HIV-1 sequences that are stored in the patient's reservoir. This effort may compensate especially if this approach can be used to cure infected individuals. A second strategy may involve the exploration of several gRNAs to target several relatively conserved sites in the HIV-1 genome in order to maximize efficacy and minimize virus escape.

Several works have demonstrated the editor specificity with reduced off-target effects and extensive potential for prophylaxis and cure of HIV-1 patients. The next steps of CRISPR-Cas-based gene therapy for HIV-1 should be further studied in clinically relevant animal models, including nonhuman primates, previous to clinical trials in humans.

## Figures and Tables

**Table 1:** Strategies and results of CRISPR-Cas9-based gene therapy for HIV-1.

Stage	Components	Target to gRNA	Method of analysis	Results	Authors
Infection (CCR5)	Cas9+3 gRNA (CR1, CR2, and CR3)	CCR5 gene	CCR5 expression	Negative cells for CCR5 expression were 10.8% (CR1), 67.7% (CR2), and 36.7% (CR3)	[35]
Infection (CCR5)	Cas9+8 gRNA (sgR5-3 and sgR5-10)	CCR5 gene	CCR5 expression	Presence of 74.1% and 63.8% of indels in the CCR5 gene	[37]
Infection (CCR5)—*in vivo*	NPG rat	—	Viral RNA quantification	Reduction of viral RNA in peripheral blood from animals that received HSPCs with edited CCR5	[38]
Infection (CCR5*Δ*32)	Cas9+gRNA	CCR5 gene	Mutations into CCR5 gene	Presence of *Δ*32 deletion in the CCR5 coreceptor and reduction of viral replication	[44]
Infection (CCR5*Δ*32)	Cas9+2 gRNA	CCR5 gene	Mutations into CCR5 gene	Presence of *Δ*32 deletion in the CCR5 coreceptor between 20% and 60% of the cells	[41]
Infection (CCR5*Δ*32)—*in vivo*	Allogeneic stem cells transplantation with CCR5Δ32 mutation	—	CXCR4 and CCR5 gene expression	Interruption of antiretrovirals and reduction to undetectable levels of viral RNA	[45]
Infection (CCR5*Δ*32)—*in vivo*	Allogeneic stem cell transplantation with CCR5*Δ*32 mutation	—	CXCR4 and CCR5 gene expression	Reduction to undetectable levels of viral charge	[46]
Infection (CXCR4)	Cas9+10 gRNA	Conserved sites of CXCR4 gene	CXCR4 expression	Reduction of CXCR4 expression by 23.5% and 45%	[48]
Infection (CXCR4)	Cas9+gRNA	CXCR4 gene	CXCR4 expression	Reduction of CXCR4 expression by 60%	[50]
Infection (CXCR4)	Cas9+2 gRNA	CXCR4 gene	CXCR4 expression	Reduction of CXCR4 expression by 18.4% and 12.0%	[49]
Infection (CXCR4 and CCR5)	Cas9+2 gRNA	CXCR4 and CCR5 gene	CXCR4 and CCR5 gene expression	Presence of indels in 40.57% of the CXCR4 gene and 32.95% of the CCR5 gene	[52]
Infection (stable immunity)	Cas9+gRNA (stable in the cellular genome)	LTR (U3 and R) and *rev*	GFP reporter gene expression	Threefold HIV-1 expression reduction and resistance to viral infection	[54]
Viral infection	Cas9 (stable in the cellular genome)+2 gRNA	LTR	Viral particles	Rupture of viral genetic material and reduction of luciferase activity	[55]
Viral infection	Cas9+2 gRNA (stable in the cellular genome)	LTR	GFP reporter gene expression	Prevention of new HIV-1 infection by immunizing cells	[56]
Viral replication	Cas9-NLS+gRNA	LTR, *gag*, *pol*, *tat*, and *rev*	YFP reporter gene and Gag expression	Reduction of 57-89% in YFP expression and decline in Gag expression	[53]
Viral replication	Cas9+3 gRNA	LTR, *gag* e *pol*	Luciferase reporter gene expression	Reduction of 23% and 96% in luciferase expression with different combinations of gRNAs	[57]
Viral replication	Cas9+gRNA	LTR	HIV-1 copy number quantification Gag and p24 expression	Reduction in HIV-1 copy number between 56% and 92% and decline in Gag and p24 expression in all patients	[55]
Viral replication	Cas9+gRNA	LTR and viral genes	p24 expression	Reduction in p24 expression	[58]
Viral replication	Cas9+gRNA	LTR and viral genes	Viral replication	Reduction of viral replication, which depends on the combinations of gRNAs used	[59]
Viral replication	Cas9+2 gRNA (stable in the cellular genome)	*Gag*, *pol*, *env*, and *rev*	Viral replication	Reduction of viral particles and number of infection cells Reduction of reverse transcriptase activity	[60]
Viral integration	Cas9+gRNA	GFP coding region and LTR	GFP reporter gene expression	Reduction in GFP expression	[62]
Viral integration	Cas9-NLS+gRNA	LTR (R and U5)	Initial, late, and integrated HIV-1 DNA	Reduction of three- to fivefold of integrated viral DNA, twofold of late DNA, and no significant change in early DNA	[53]
Latency	Cas9+2 gRNA	LTR (TAR and NF-*κ*B)	GFP reporter gene expression and MIF	Reduction in GFP and MIF expression for up to 20.0% potentiated after some transductions	[62]
Latency	Cas9+gRNA	GFP coding region and LTR	GFP reporter gene expression	Reduction of GFP expression, regardless of the amount of integrated virus, potentiated after some transductions	[54]
Latency	Cas9+10 gRNA	LTR, *pol*, *tat*, and *rev*	Activation of viral expression by GFP and p24	Reduction of 3- to 10-fold of GFP expression and 20-fold of p24 expression	[63]
Latency	Cas9+2 gRNA	LTR	GFP reporter gene expression	Reduction of GFP-positive cells to 4.5%	[62]
Latency	Cas9+4 gRNA	LTR (U3 region)	GFP reporter gene expression	Reduction of GFP and elimination of the proviral fragment, preventing reactivation and viral replication	[56]
Latency	Cas9+2 gRNA	—	GFP reporter gene expression and viral mRNA	Reduction of GFP and viral mRNA expression	[38]
Latency	Cas9+gRNA	*gag*, *env*, *pol*, *vif*, *rev*, and LTR	GFP reporter gene expression	Reduction in GFP expression between 48% and 92%, potentiated with multiple transductions	[62]
Latency	Cas9+43 gRNA	*gag*, *env*, *pol*, *vif*, *rev*, and LTR	P24 expression	Reduction of p24 expression, potentiated with multiple transductions	[62]
Latency (*in vivo*)	Cas9+2 gRNA Tg26 transgenic mice	LTR and *gag*	DNA extracted from the liver lymphocytes, heart, spleen, lung, kidney, brain and blood	Presence of a 1323 bp and 368 bp fragments, with an additional fragment of 183 bp	[65]
Latency (*in vivo*)	Cas9+2 gRNA Rats	LTR and *gag*	DNA extracted from liver, lymphocytes, heart, spleen, lung, kidney, brain, and blood	Elimination of viral fragment and reduction of viral mRNA	[65]
Latency (*in vivo*)	Cas9+gRNA NCr nude mouse BLT humanized mice	LTR and *gag*	Rupture of genomic DNA in several organs and tissues	Reduction of viral expression Fragmentary deletion of provirus in several organs and tissues in the LTR and *gag* regions	[68]
Latency (*in vivo*)	Cas9+2 gRNA NRG rats	LTR	Viral DNA	Reduction in more 90% of viral DNA and elimination of viral fragment present between target sites	[70]

## References

[1] Seitz R. (2016). Human immunodeficiency virus (HIV). *Transfusion Medicine and Hemotherapy*.

[2] Shaw G. M., Hunter E. (2012). HIV transmission. *Cold Spring Harbor Perspectives in Medicine*.

[3] Rumbwere Dube B. N., Marshall T. P., Ryan R. P. (2016). Predictors of human immunodeficiency virus (HIV) infection in primary care: a systematic review protocol. *Systematic Reviews*.

[4] World Health Organization (2018). *HIV/AIDS: Data and Statistics*.

[5] World Health Organization (2018). *HIV/AIDS: Signs and Symptoms*.

[6] Montaner J. S. G., Lima V. D., Barrios R. (2010). Association of highly active antiretroviral therapy coverage, population viral load, and yearly new HIV diagnoses in British Columbia, Canada: a population-based study. *The Lancet*.

[7] Maartens G., Celum C., Lewin S. R. (2014). HIV infection: epidemiology, pathogenesis, treatment, and prevention. *The Lancet*.

[8] Wainberg M. A., Zaharatos G. J., Brenner B. G. (2011). Development of antiretroviral drug resistance. *The New England Journal of Medicine*.

[9] Desai M., Dikshit R. K., Iyer G. (2012). Antiretroviral drugs: critical issues and recent advances. *Indian Journal of Pharmacology*.

[10] Strayer D. S., Akkina R., Bunnell B. A. (2005). Current status of gene therapy strategies to treat HIV/AIDS. *Molecular Therapy*.

[11] Hsu P. D., Lander E. S., Zhang F. (2014). Development and applications of CRISPR-Cas9 for genome engineering. *Cell*.

[12] Fanales-Belasio E., Raimondo M., Suligoi B., Buttò S. (2010). HIV virology and pathogenetic mechanisms of infection: a brief overview. *Annali dell'Istituto Superiore di Sanita*.

[13] Feng Y., Broder C. C., Kennedy P. E., Berger E. A. (1996). HIV-1 entry cofactor: functional cDNA cloning of a seven-transmembrane, G protein-coupled receptor. *Science*.

[14] Deeks S. G., Overbaugh J., Phillips A., Buchbinder S. (2015). HIV infection. *Nature Reviews Disease Primers*.

[15] Freed E. O. (2015). HIV-1 assembly, release and maturation. *Nature Reviews Microbiology*.

[16] Park I. W., Han C., Song X. (2009). Inhibition of HIV-1 entry by extracts derived from traditional Chinese medicinal herbal plants. *BMC Complementary and Alternative Medicine*.

[17] Williamson S. (2003). Adaptation in the env gene of HIV-1 and evolutionary theories of disease progression. *Molecular Biology and Evolution*.

[18] O’Brien S. J., Moore J. P. (2000). The effect of genetic variation in chemokines and their receptors on HIV transmission and progression to AIDS. *Immunological Reviews*.

[19] Doms R. W. (2001). Chemokine receptors and HIV entry. *AIDS*.

[20] Rose J. D., Rhea A. M., Weber J., Quiñones-Mateu M. E. (2009). Current tests to evaluate HIV-1 coreceptor tropism. *Current Opinion in HIV and AIDS*.

[21] Mojica F. J. M., Ferrer C., Juez G., Rodríguez-Valera F. (1995). Long stretches of short tandem repeats are present in the largest replicons of the archaea Haloferax mediterranei and Haloferax volcanii and could be involved in replicon partitioning. *Molecular Microbiology*.

[22] Mojica F. J. M., Díez-Villaseñor C., Soria E., Juez G. (2000). Biological significance of a family of regularly spaced repeats in the genomes of archaea, bacteria and mitochondria. *Molecular Microbiology*.

[23] Jansen R., Embden J. D. A., Gaastra W., Schouls L. M. (2002). Identification of genes that are associated with DNA repeats in prokaryotes. *Molecular Microbiology*.

[24] Haft D. H., Selengut J., Mongodin E. F., Nelson K. E. (2005). A guild of 45 CRISPR-associated (Cas) protein families and multiple CRISPR/Cas subtypes exist in prokaryotic genomes. *PLoS*.

[25] Mojica F. J. M., Díez-Villaseñor C., García-Martínez J., Soria E. (2005). Intervening sequences of regularly spaced prokaryotic repeats derive from foreign genetic elements. *Journal of Molecular Evolution*.

[26] Pourcel C., Salvignol G., Vergnaud G. (2005). CRISPR elements in Yersinia pestis acquire new repeats by preferential uptake of bacteriophage DNA, and provide additional tools for evolutionary studies. *Microbiology*.

[27] Brouns S. J. J., Jore M. M., Lundgren M. (2008). Small CRISPR RNAs guide antiviral defense in prokaryotes. *Science*.

[28] Marraffini L. A., Sontheimer E. J. (2018). CRISPR interference limits horizontal gene transfer in staphylococci by targeting DNA. *Science*.

[29] Deltcheva E., Chylinski K., Sharma C. M. (2011). CRISPR RNA maturation by trans-encoded small RNA and host factor RNase III. *Nature*.

[30] Jinek M., Chylinski K., Fonfara I., Hauer M., Doudna J. A., Charpentier E. (2012). A programmable dual-RNA-guided DNA endonuclease in adaptive bacterial immunity. *Science*.

[31] Karvelis T., Gasiunas G., Young J. (2015). Rapid characterization of CRISPR-Cas9 protospacer adjacent motif sequence elements. *Genome Biology*.

[32] Sternberg S. H., Redding S., Jinek M., Greene E. C., Doudna J. A. (2014). DNA interrogation by the CRISPR RNA-guided endonuclease Cas9. *Nature*.

[33] Gilbert L. A., Larson M. H., Morsut L. (2013). CRISPR-mediated modular RNA-guided regulation of transcription in eukaryotes. *Cell*.

[34] Alkhatib G., Combadiere C., Broder C. C. (1996). CC CKR5: a RANTES, MIP-1*α*, MIP-1*β*, receptor as a fusion cofactor for macrophage-tropic HIV-1. *Science*.

[35] Wang W., Ye C., Liu J., Zhang D., Kimata J. T., Zhou P. (2014). CCR5 gene disruption via lentiviral vectors expressing Cas9 and single guided RNA renders cells resistant to HIV-1 infection. *PLoS One*.

[36] Lopalco L. (2010). CCR5: from natural resistance to a new anti-HIV strategy. *Viruses*.

[37] Griffin G. E., Liu Y., Li C. (2015). Inhibition of HIV-1 infection of primary CD4^+^ T-cells by gene editing of CCR5 using adenovirus-delivered CRISPR/Cas9. *Journal of General Virology*.

[38] Xu L., Yang H., Gao Y. (2017). CRISPR/Cas9-mediated CCR5 ablation in human hematopoietic stem/progenitor cells confers HIV-1 resistance in vivo. *Molecular Therapy*.

[39] Samson M., Libert F., Doranz B. J. (1996). Resistance to HIV-1 infection in Caucasian individuals bearing mutant alleles of the CCR-5 chemokine receptor gene. *Nature*.

[40] Liu R., Paxton W. A., Choe S. (1996). Homozygous defect in HIV-1 coreceptor accounts for resistance of some multiply-exposed individuals to HIV-1 infection. *Cell*.

[41] Qi C., Li D., Jiang X. (2018). Inducing CCR5Δ32/Δ32 homozygotes in the human Jurkat CD4^+^ cell line and primary CD4^+^ cells by CRISPR-Cas9 genome-editing technology. *Molecular Therapy - Nucleic Acids*.

[42] Ellwanger J. H., Leal B. K., Valverde-Villegas J. M. (2018). CCR5Δ32 in HCV infection, HCV/HIV co-infection, and HCV-related diseases. *Infection, Genetics and Evolution*.

[43] Matos A. R., Martins J. S. C. C., Oliveira M. L. A., Garcia C. C., Siqueira M. M. (2019). Human CCR5Δ32 (rs333) polymorphism has no influence on severity and mortality of influenza A(H1N1)pdm09 infection in Brazilian patients from the post pandemic period. *Infection, Genetics and Evolution*.

[44] Ye L., Wang J., Beyer A. I. (2014). Seamless modification of wild-type induced pluripotent stem cells to the natural CCR5Δ32 mutation confers resistance to HIV infection. *Proceedings of the National Academy of Sciences of the United States of America*.

[45] Gupta R. K., Abdul-Jawad S., McCoy L. E. (2019). HIV-1 remission following CCR5Δ32/Δ32 haematopoietic stem-cell transplantation. *Nature*.

[46] Hütter G., Nowak D., Mossner M. (2009). Long-term control of HIV by CCR5Delta32/Delta32 stem-cell transplantation. *New England Journal of Medicine*.

[47] Contento R. L., Molon B., Boularan C. (2008). CXCR4–CCR5: a couple modulating T cell functions. *Proceedings of the National Academy of Sciences*.

[48] Hou P., Chen S., Wang S. (2015). Genome editing of CXCR4 by CRISPR/Cas9 confers cells resistant to HIV-1 infection. *Scientific Reports*.

[49] Liu S., Wang Q., Yu X. (2018). HIV-1 inhibition in cells with CXCR4 mutant genome created by CRISPR-Cas9 and piggyBac recombinant technologies. *Scientific Reports*.

[50] Schumann K., Lin S., Boyer E. (2015). Generation of knock-in primary human T cells using Cas9 ribonucleoproteins. *Proceedings of the National Academy of Sciences of the United States of America*.

[51] Tian S., Choi W. T., Liu D. (2005). Distinct functional sites for human immunodeficiency virus type 1 and stromal cell-derived factor 1*α* on CXCR4 transmembrane helical domains. *Journal of Virology*.

[52] Liu Z., Chen S., Jin X. (2017). Genome editing of the HIV co-receptors CCR5 and CXCR4 by CRISPR-Cas9 protects CD4^+^ T cells from HIV-1 infection. *Cell & Bioscience*.

[53] Yin L., Hu S., Mei S. (2018). CRISPR/Cas9 inhibits multiple steps of HIV-1 infection. *Human Gene Therapy*.

[54] Liao H.-K., Gu Y., Diaz A. (2015). Use of the CRISPR/Cas9 system as an intracellular defense against HIV-1 infection in human cells. *Nature Communications*.

[55] Kaminski R., Chen Y., Salkind J. (2016). Negative feedback regulation of HIV-1 by gene editing strategy. *Scientific Reports*.

[56] Hu W., Kaminski R., Yang F. (2014). RNA-directed gene editing specifically eradicates latent and prevents new HIV-1 infection. *Proceedings of the National Academy of Sciences of the United States of America*.

[57] Yin C., Zhang T., Li F. (2016). Functional screening of guide RNAs targeting the regulatory and structural HIV-1 viral genome for a cure of AIDS. *AIDS*.

[58] Wang G., Zhao N., Berkhout B., Das A. T. (2016). CRISPR-Cas9 can inhibit HIV-1 replication but NHEJ repair facilitates virus escape. *Molecular Therapy*.

[59] Lebbink R. J., de Jong D. C. M., Wolters F. (2017). A combinational CRISPR/Cas9 gene-editing approach can halt HIV replication and prevent viral escape. *Scientific Reports*.

[60] Wang Z., Pan Q., Gendron P. (2016). CRISPR/Cas9-derived mutations both inhibit HIV-1 replication and accelerate viral escape. *Cell Reports*.

[61] Ebina H., Misawa N., Kanemura Y., Koyanagi Y. (2013). Harnessing the CRISPR/Cas9 system to disrupt latent HIV-1 provirus. *Scientific Reports*.

[62] Wang Q., Liu S., Liu Z. (2018). Genome scale screening identification of SaCas9/gRNAs for targeting HIV-1 provirus and supression of HIV-1 infection. *Virus Research*.

[63] Zhu W., Lei R., le Duff Y. (2015). The CRISPR/Cas9 system inactivates latent HIV-1 proviral DNA. *Retrovirology*.

[64] Kunze C., Börner K., Kienle E. (2018). Synthetic AAV/CRISPR vectors for blocking HIV-1 expression in persistently infected astrocytes. *Glia*.

[65] Kaminski R., Bella R., Yin C. (2016). Excision of HIV-1 DNA by gene editing: a proof-of-concept in vivo study. *Gene Therapy*.

[66] Soriano V. (2017). Hot news: gene therapy with CRISPR/Cas9 coming to age for HIV cure. *AIDS Reviews*.

[67] De Silva Feelixge H. S., Jerome K. R. (2017). Excision of latent HIV-1 from infected cells in vivo: an important step forward. *Molecular Therapy*.

[68] Yin C., Zhang T., Qu X. (2017). In vivo excision of HIV-1 provirus by saCas9 and multiplex single-guide RNAs in animal models. *Molecular Therapy*.

[69] Karpel M. E., Boutwell C. L., Allen T. M. (2015). BLT humanized mice as a small animal model of HIV infection. *Current Opinion in Virology*.

[70] Bella R., Kaminski R., Mancuso P. (2018). Removal of HIV DNA by CRISPR from patient blood engrafts in humanized mice. *Molecular Therapy - Nucleic Acids*.

[71] Kleinstiver B. P., Pattanayak V., Prew M. S. (2016). High-fidelity CRISPR-Cas9 nucleases with no detectable genome-wide off-target effects. *Nature*.

[72] Kulcsár P. I., Tálas A., Huszár K. (2017). Crossing enhanced and high fidelity SpCas9 nucleases to optimize specificity and cleavage. *Genome Biology*.

[73] Vakulskas C. A., Dever D. P., Rettig G. R. (2018). A high-fidelity Cas9 mutant delivered as a ribonucleoprotein complex enables efficient gene editing in human hematopoietic stem and progenitor cells. *Nature Medicine*.

[74] Hsu P. D., Scott D. A., Weinstein J. A. (2013). DNA targeting specificity of RNA-guided Cas9 nucleases. *Nature Biotechnology*.

[75] Listgarten J., Weinstein M., Kleinstiver B. P. (2018). Prediction of off-target activities for the end-to-end design of CRISPR guide RNAs. *Nature Biomedical Engineering*.

[76] Haeussler M., Schönig K., Eckert H. (2016). Evaluation of off-target and on-target scoring algorithms and integration into the guide RNA selection tool CRISPOR. *Genome Biology*.

[77] Hendel A., Bak R. O., Clark J. T. (2015). Chemically modified guide RNAs enhance CRISPR-Cas genome editing in human primary cells. *Nature Biotechnology*.

[78] Basila M., Kelley M. L., Smith A. . B. (2017). Minimal 2′-O-methyl phosphorothioate linkage modification pattern of synthetic guide RNAs for increased stability and efficient CRISPR-Cas9 gene editing avoiding cellular toxicity. *PLoS One*.

[79] Senturk S., Shirole N. H., Nowak D. G. (2017). Rapid and tunable method to temporally control gene editing based on conditional Cas9 stabilization. *Nature Communications*.

[80] Liang X., Potter J., Kumar S. (2015). Rapid and highly efficient mammalian cell engineering via Cas9 protein transfection. *Journal of Biotechnology*.

[81] Cho S. W., Kim S., Kim J. M., Kim J. S. (2013). Targeted genome engineering in human cells with the Cas9 RNA-guided endonuclease. *Nature Biotechnology*.

[82] Cho S. W., Kim S., Kim Y. (2014). Analysis of off-target effects of CRISPR/Cas-derived RNA-guided endonucleases and nickases. *Genome Research*.

[83] Ran F. A., Hsu P. D., Lin C. Y. (2013). Double nicking by RNA-guided CRISPR Cas9 for enhanced genome editing specificity. *Cell*.

[84] Pattanayak V., Lin S., Guilinger J. P., Ma E., Doudna J. A., Liu D. R. (2013). High-throughput profiling of off-target DNA cleavage reveals RNA-programmed Cas9 nuclease specificity. *Nature Biotechnology*.

[85] Shen B., Zhang W., Zhang J. (2014). Efficient genome modification by CRISPR-Cas9 nickase with minimal off-target effects. *Nature Methods*.

[86] Zhang X. H., Tee L. Y., Wang X. G., Huang Q. S., Yang S. H. (2015). Off-target effects in CRISPR/Cas9-mediated genome engineering. *Molecular Therapy - Nucleic Acids*.

[87] Krimsky S. (2019). Ten ways in which He Jiankui violated ethics. *Nature Biotechnology*.

[88] Evitt N. H., Mascharak S., Altman R. B. (2015). Human germline CRISPR-Cas modification: toward a regulatory framework. *The American Journal of Bioethics*.

[89] Li J. R., Walker S., Nie J. B., Zhang X. Q. (2019). Experiments that led to the first gene-edited babies: the ethical failings and the urgent need for better governance. *Journal of Zhejiang University-SCIENCE B*.

[90] Committee on Science, Technology, and Law Policy and Global Affairs (2015). *International Summit on Human Gene Editing: A Global Discussion*.

[91] Pollack R. (2015). Eugenics lurk in the shadow of CRISPR. *Science*.

